# Preassessment Interview Improves the Efficacy and Safety of Bowel Preparation for Colonoscopy

**DOI:** 10.1155/2016/7591637

**Published:** 2016-11-27

**Authors:** Hari Padmanabhan, Alexander Rothnie, Andy Higgins, Amandeep Grewal, Katherine Arndtz, Alan. M. Nevill, Matthew. J. Brookes, Ray Mathew

**Affiliations:** ^1^Gastroenterology Unit, County Hospital, Stafford, UK; ^2^Research Institute of Healthcare Sciences, University of Wolverhampton, Wolverhampton, UK; ^3^Gastroenterology Unit, New Cross Hospital, Wolverhampton, UK

## Abstract

*Aim*. To determine whether preassessment improves bowel preparation quality and prevents renal deterioration for chronic kidney disease (CKD) patients.* Methods*. Data was collected prospectively starting in January 2011 for 12 months. Patients were divided according to the presence or absence of preassessment and stratified to one of three risk groups based on patient's comorbidities and identified risk factors for poor bowel preparation; group 1 had no risk factors, group 2 had 1 risk factor, and group 3 patients had 2 or more risk factors. The association between preassessment and bowel preparation quality was analyzed using binary logistic regression.* Results*. 1840 colonoscopies were carried out during the period. Total number analyzed was 1704. 404 patients were preassessed. Preassessment patients had significantly better bowel preparation across all groups (OR 1.605; *p* = 0.002). Group 3 patients were 52% more likely to have good bowel preparation (*p* = 0.04) if they had been preassessed. Eighty-eight patients were identified with an eGFR < 60 mL/min. There was a significant difference in the eGFR percentage change between patients with preassessment and those without (*p* = 0.006).* Conclusions*. Face-to-face preassessment appears to improve the quality of bowel preparation and aids in minimizing the risk of renal injury in patients with CKD.

## 1. Introduction

Oral bowel cleansing agents (OBCAs) are widely used before endoscopic and radiological assessment of the bowel and preoperatively to aid with diagnostic assessment and minimizing fecal contamination [1]. For the most part, these agents are well tolerated, safe, and efficacious. However, there is evidence that bowel cleansing can potentially cause significant harm to the patient.

In 2009, the National Patient Safety Agency (NPSA) issued an alert regarding the use of OBCAs [2]. This report cited 1 death and detailed 218 patient safety incidents directly attributable to the use of oral bowel preparations, including electrolyte abnormalities, dehydration, and renal failure. It highlighted certain cohorts of patients at particularly high risk: the frail, elderly, children, and patients with contraindications to bowel preparations (such as bowel obstruction or the presence of an ileostomy). This led to the Central Alerting System of the UK Department of Health to issue directives to all NHS trusts regarding the prescription of bowel preparations and the patient assessment required for safe administration [2].

Consequently, consensus guidelines were developed in late 2009, which were later revised and published in 2012 [1]. These guidelines suggest that a screening process is necessary to ensure that “at-risk” patients are identified and prepared appropriately. At-risk patients include those with specific comorbidities, such as patients with chronic kidney disease (CKD), congestive cardiac failure (CCF), liver cirrhosis or ascites, patients on dialysis, and prescription of certain medications (including diuretics, nonsteroidal anti-inflammatory drugs [NSAIDs], and angiotensin-converting-enzyme [ACE] inhibitors). The consensus guidelines recommend a clinical evaluation, measurement of serum eGFR, and a review for the above medications and comorbidities. Based on these parameters, a recent study suggested that up to 64% of patients could be defined as “at risk” of adverse events from bowel preparation [3].

NHS trusts in the UK have responded to these guidelines with varying approaches and as such changes have a significant impact on resources. One way of implementing such guidelines has been with the use of a preassessment clinic. Organizing a specific precolonoscopy assessment, either by telephone or in person, has been shown to be beneficial in reducing nonattendance for the procedure itself [4]. As yet, only a few studies have investigated the effects of these preassessments on both the safety and efficacy of bowel preparation.

The aim of this study was to assess how preassessment of patients prior to colonoscopy affects a number of factors concerned with the procedure itself, including the quality of bowel preparation, the effect on eGFR, and the use of a risk-stratifying approach, to deploy resources for those patients most at risk of poor bowel preparation.

## 2. Materials and Methods

This was an observational, retrospective study of prospectively collected data from our endoscopy database. Data were collected over a period of 12 months starting in January 2011; preassessment began at our NHS trust in September 2011. This study was approved by the local research authority, and the need for informed consent from the patients was waived.

Assessment was made in keeping with the 4 recommendations made by the NPSA: (1) clinical assessment prior to prescription of bowel preparation, (2) authorization of bowel preparation by clinical staff, (3) an explanation of the bowel preparation to every patient, and (4) dispensation of the medication by authorized professionals with verbal and written explanation [2]. All patients requiring an outpatient colonoscopy received a face-to-face interview with a trained nurse. A strict proforma was used in order to ensure that all aspects of the procedure and any coexisting medical conditions or medications were considered. If no concerns were raised, then the bowel preparation, along with verbal and written information regarding appropriate self-administration, was given to the patient. However, if concerns arose during the preassessment, then the nurse was responsible for contacting a gastroenterology consultant for further advice and management. Depending on the patient, the gastroenterology consultant suggested a change in medication, cancellation of the procedure in favour of an alternative test, or even organizing a hospital admission to ensure patient safety. The latter was considered in elderly patients with limited mobility who were unlikely to be able to cope with the bowel preparation at home, but for whom no alternative test was available. Additionally, patients with CKD who had an eGFR < 30 mL/min were hospitalized, such that renal function could be closely monitored, with intravenous fluids given as required.

Patients who underwent colonoscopy at our unit were divided into 2 groups, based on whether they received preassessment; patients between January and August were not preassessed, while the majority who were seen between September and December did receive preassessment. All patients from both groups were retrospectively assessed for “at-risk” conditions; “at-risk” patients included those on regular medication considered unsafe by the NPSA and consensus guidelines when administering OBCAs (NSAIDs and diuretics), patients with comorbidities (CKD, CCF, cirrhosis, and diabetes), and those who were “at risk” of poor bowel preparation (constipation and limited mobility). A proforma, based on NPSA recommendations, was used to stratify the study group (*n* = 1840) into 3 risk groups, based on the presence of “at-risk” conditions. Patients in group 1 had no risk factors, while those in group 2 had at least 1 risk factor, and those in group 3 had 2 or more risk factors.

The primary end point was successful bowel preparation which was an objective assessment using the Aronchick bowel preparation scale [5]. This scale enables rating the whole colon with a single score and defines a good preparation as visualizing >90% of the mucosa. Patients with CKD had eGFR checked before and within 1 week after administration of OBCA. A “low eGFR” was defined as <60 mL/min. All colonoscopies were performed in a dedicated endoscopy unit by gastroenterology or surgical consultants and specialist endoscopy nurses. The standard oral bowel preparation regime was 4 sachets of polyethylene glycol (Kleanprep, Norgine Ltd., Middlesex, UK). Kleanprep is diluted in 1 L of water. Participants undergoing morning procedures received day-before bowel preparation on the day prior to the colonoscopy with instructions to fast from 1400 h, take first sachet at 1600 h, and then continue with the following three sachets until bedtime. Participants undergoing afternoon procedures were asked to fast from 1800 h on the day prior to colonoscopy and then take three sachets starting at 1800 h and the other one sachet the following morning before 0800 h. Complete colonoscopy was defined as visualization and intubation of the caecum, confirmed by identification of the ileocecal valve and triradiate fold.

Continuous variables are expressed as mean (standard deviation) or median; group comparisons were carried out using the* t*-test or Mann–Whitney* U* test, as appropriate. Categorical variables are expressed as percentages and were analyzed using the chi-square (*χ* ^2^) test. A* p* value ≤ 0.05 was considered significant for all statistical tests. The effect of preassessment (with or without) on the quality of bowel preparation was analyzed initially for all 3 groups combined and then for each group separately, using binary logistic regression. All statistical tests were performed using SPSS (SPSS 15, Chicago, IL).

## 3. Results

During the study period, 1840 colonoscopies were performed. Patients were omitted when there was no clear comment on the quality of bowel preparation in the report, giving a final study cohort of 1704 patients. The mean age was 61.7 years (range 16–94). A total of 404 patients received preassessment. With respect to the quality of bowel preparation, 79.5% (*n* = 1354) of patients had good bowel preparation, while 20.5% had poor bowel preparation (*n* = 350). Patient demographic characteristics are shown in Table 1.

Preassessment significantly increased the quality of bowel preparation across all groups (OR 1.605; *p* = 0.002). In groups 1 and 2, the likelihood of having a good quality bowel preparation was 80% and 72% higher, respectively, in patients who received preassessment; however, these improvements did not reach statistical significance (Table 2). Patients stratified into group 3 who received preassessment were 52% more likely to have good bowel preparation (*p* = 0.039) than those who were not preassessed. Age and sex were not shown to affect the quality of bowel preparation in our study.

We examined the reasons for an incomplete colonoscopy (Table 3). A greater risk of incomplete colonoscopy was observed in patients with poor bowel preparation (*n* = 81; *p* = 0.006).

Additionally, we looked at the interventions (Table 4) that were performed in the preassessment group (*n* = 404). 9.7% (*n* = 39) of the patients within the preassessment group were discussed with the gastroenterologist in view of significant concerns. Out of 28 patients with CKD who underwent preassessment, 12 patients (eGFR < 30 mL/min; 2.9%) were hospitalized, such that renal function could be closely monitored. To prevent deterioration in eGFR and to improve quality of bowel preparation, 4.6% of patients (*n* = 20) had alteration to their medications. Extrabowel preparation was given to 6.7% (*n* = 27) of patients with history of severe constipation.

**Figure 1:**
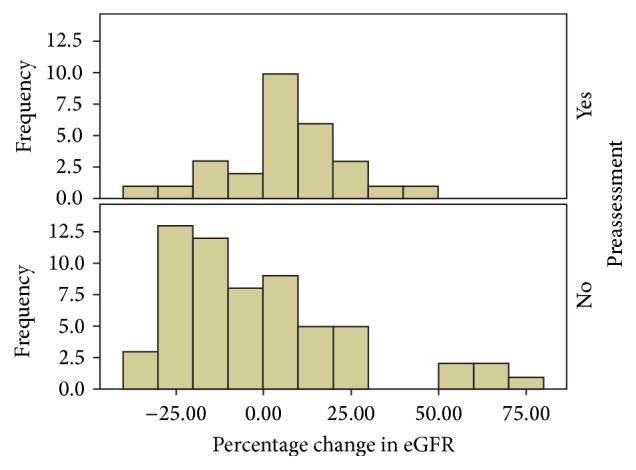
Percentage change in eGFR in patients with or without preassessment. eGFR: estimated glomerular filtration rate.

Eighty-eight patients had an eGFR < 60 mL/min. Of these patients, there was a significant difference in the percentage change in eGFR from pre- to postadministration of OBCA between those patients who had preassessment (median = 7.7%) and those who did not (median = −6.6%) (*p* = 0.006, Mann–Whitney) (Figure 1).

## 4. Discussion

Our study suggests that face-to-face assessment prior to colonoscopy, as recommended by the consensus guidelines for prescription and administration of OBCAs, improves the quality of bowel preparation across all patients. Most notably, patients stratified into group 3, the “high-risk” group, exhibited a statistically significant improvement (*p* = 0.039) in the quality of bowel preparation. This cohort was considered to be at high risk because of the presence of multiple comorbidities, which appear to affect the quality of bowel preparation and may ultimately lead to failed and/or repeat procedures. Preassessment in this group facilitates specific modification of medications and bowel OBCAs to optimize the chance of a successful procedure. While the likelihood of good bowel preparation improved with preassessment in groups 1 and 2, no statistical significance was observed. One could argue that these groups, and especially group 1, have the least to gain from drug alterations or modification of OBCAs, due to a comparatively lower initial risk. As such, this study may not be adequately designed to determine any significant benefits for groups 1 and 2.

We have also shown that preassessment allows close monitoring of patients with CKD and specific alteration of their medications and OBCAs accordingly. Consequently, we have shown that eGFR did not decline with administration of OBCA, potentially minimizing the risk of renal injury.

Good quality bowel preparation has been noted as a key factor in the performance of a high quality colonoscopy and improving polypectomy rates [6]. Studies have shown that up to 25% of colonoscopies experience inadequate bowel preparation, resulting in cautious interpretation of colonoscopy results [6, 7]. This has substantial impact on the procedural duration, difficulty, completion, and cost [6–11]. Many factors can affect this quality, including the patient's mobility, medications, and any preexisting constipation. During the preassessment process, these factors are assessed, and changes to the bowel preparation can be made. Patients having risk factors for poor quality bowel preparation can be prescribed additional bowel preparation; however, this needs to be balanced against the risk of toxicity on an individual basis.

A face-to-face precolonoscopy consultation helps to obtain information on patient's comorbidities, medication use, health status, and the need for any specific precaution [12]. Using criteria identified by the consensus guidelines for prescription and administration of OBCAs, a risk-stratification model of preexamination risk factors may help to identify patients at significant risk. Our results are very encouraging; we have demonstrated that face-to-face preassessment not only improves the quality of bowel preparation but also reduces the risk of renal injury.

Further development of this risk-stratification model could involve the introduction of a telephone assessment for “low-risk” patients (i.e., those who fall into group 1). In the context of the fecal occult blood test colorectal cancer-screening program, Rodger and Steele found that when patients were given the option of a telephone interview rather than a face-to-face consultation, nonattendance rates fell from 14.9% to 0.8% [13]. Of this cohort, 97% of patients who underwent a telephone interview found the process acceptable, and 93% found it a positive experience. Stoop et al. studied a larger data set of 6600 patients who were invited to participate in the colorectal cancer-screening program [12]. The number of patients who chose to participate in either a telephone interview or face-to-face consultation was comparable; however, colonoscopy participation was significantly lower in the group who had been interviewed over the telephone. They also found that the expected pain of bowel preparation was significantly higher in the telephone interview group, while factors such as expected embarrassment, pain, and burden of the colonoscopy itself were similar for both groups. Both the aforementioned studies examined the adequacy of bowel preparation and found no difference between the telephone and face-to-face consultation groups.

With this in mind, the possibility of telephone preassessment looks promising. By streamlining our service, we have the opportunity to save selected low-risk patients time and money, while simultaneously improving the quality of the colonoscopy through telephone preassessment. By freeing up resources at the hospital, more time and attention can be dedicated to patients with greater risk, resulting in better planning, reduced risk of harm to patients, and increased efficiency of the hospital's service. Further work is necessary in this field, specifically related to patient satisfaction, attendance rates, and cost effectiveness.

Limitations of our study include the retrospective analysis of clinic letters, which occasionally lacked crucial data, including information about CKD and medication history. This may have led to a bias of Type 2 error and could have underreported the presence of comorbidities. Thus, in some cases, patients may have been wrongly grouped into lower risk groups. There are patients between September and December who have been allocated into the group who had no preassessment. This may have led to a selection bias in this study. There could have been few uncontrolled factors which might have affected the results such as patient education, alteration of medications by gastroenterologists, and timing of procedure (morning versus afternoon) and body mass index. These factors were not described in the current study. The risk-stratification model is limited to some extent in that it was not validated. Additionally, the measurement of eGFR was rarely consistent with respect to timing, which may have altered the observed percentage change in eGFR.

Our study has shown that face-to-face preassessment improves the quality of bowel preparation for patients undergoing colonoscopy, with the greatest benefit observed in “at-risk” patients who are at a high risk of poor bowel preparation. We have demonstrated that preassessment significantly reduces the fall in eGFR for those with CKD and helps minimize the risk of renal injury. We conclude that preassessment is a prerequisite for patients who are at risk of poor bowel preparation and with significant comorbidities. Further studies regarding the potential for telephone assessment of low-risk patients may highlight further opportunities to improve patient safety and resource allocation.

## Figures and Tables

**Table 1:** Patient demographic characteristics.

Total colonoscopies	1840
Total number analyzed	1704
Patients preassessed	404
Mean age	61.7 years
“At-risk” medication	36.8% (*n* = 677)
“At-risk” comorbidities	11% (*n* = 202)
“At risk” of poor bowel preparation	17.2% (*n* = 318)
Patients with low eGFR (<60 mL/min)	88

eGFR: estimated glomerular filtration rate.

**Table 2:** The effect of preassessment on the quality of bowel preparation for risk groups, using binary logistic regression.

Risk groups	Number of patients	Percentage	Preassessment		*p* value	Odds ratio
			Yes	No		
Group 1	258	15.1	65	193	0.158	1.80
Group 2	607	35.6	141	466	0.063	1.79
Group 3	839	49.2	198	641	0.039	1.52
All groups	1704		404	1300	0.002	1.61
CKD	88		28	60	0.006 (Mann–Whitney)	

CKD: chronic kidney disease.

**Table 3:** Reasons for incomplete colonoscopy, according to risk group.

		Reasons for incomplete colonoscopy						Total
		Technically difficult	Impassable stricture	Poor bowel preparation	Procedure abandoned	Pain	Colitis	
Group stratification	Group 1	4	1	9	0	0	1	15
	Group 2	11	7	18	2	3	0	41
	Group 3	21	17	54	0	4	0	96

Total		36	25	81	2	7	1	152

**Table 4:** Interventions undertaken during preassessment.

Interventions	Number	Percentage
TCI	12	2.9%
Medication adjusted	20	4.6%
Extra preparation	27	6.7%
Consultant review	39	9.7%

TCI: “to come in” to hospital.
